# A Model Eumelanin from 5,6-Dihydroxyindole-2-Carboxybutanamide Combining Remarkable Antioxidant and Photoprotective Properties with a Favourable Solubility Profile for Dermo-Cosmetic Applications

**DOI:** 10.3390/ijms24044241

**Published:** 2023-02-20

**Authors:** Rita Argenziano, Maria Laura Alfieri, Noemi Gallucci, Gerardino D’Errico, Lucia Panzella, Alessandra Napolitano

**Affiliations:** Department of Chemical Sciences, University of Naples “Federico II”, Via Cintia 4, I-80126 Naples, Italy

**Keywords:** melanins, 5,6 dihydroxyindole, carboxybutanamide, antioxidant, photoprotection

## Abstract

The search for new synthetic melanin-related pigments that maintain the antioxidant and photoprotective properties of naturally occurring dark eumelanins, while overcoming their unfavorable solubility, and molecular heterogeneity is presently a very active issue for dermo-cosmetic purposes. In this work, we explored the potential of a melanin obtained from the carboxybutanamide of a major eumelanin biosynthetic precursor, 5,6-dihydroxyindole-2-carboxylic acid (DHICA), by aerobic oxidation under slightly alkaline conditions. Analysis of the pigment by EPR, ATR-FTIR and MALDI MS indicated a substantial structural similarity to DHICA melanin, while investigation of the early intermediates confirmed unchanged regiochemistry of the oxidative coupling. The pigment exhibited a UVA–visible absorption even more intense than that of DHICA melanin, and a noticeable solubility in polar solvents of dermo-cosmetic relevance. The hydrogen- and/or electron-donor ability, and the iron (III) reducing power as determined by conventional assays provided evidence for marked antioxidant properties not merely ascribable to the more favorable solubility profile, while the inhibitory action of the radical- or photosensitized solar light-induced lipid peroxidation was more marked compared to that of DHICA melanin. Overall, these results hint at this melanin, which remarkable properties are, in part, due to the electronic effects of the carboxyamide functionality as a promising functional ingredient for dermo-cosmetic formulations.

## 1. Introduction

Eumelanins, the dark variant of melanin pigments, are responsible for the black to brown coloring of the skin, hair and eyes of humans and mammals. The structural investigation of these pigments has represented a major challenge in natural product chemistry for decades mainly because of the unfavorable chemico-physical properties, the molecular heterogeneity and the low amounts isolable from the natural sources [1,2,3]. In recent years, interest in these pigments have shifted from investigation of their functional role in the producing organisms to rationalization of the structure-properties relationship [4,5,6,7,8] in order to define the key elements for obtaining melanin related materials, including polydopamine from dopamine oxidation, with specific features for application in biomedical and related fields, such as bioelectronics [9], surface functionalization [10] and dermo-cosmetics [11].

A series of studies carried out on model melanin pigments prepared by oxidation of the ultimate monomeric precursors, namely 5,6-dihydroxyindole (DHI) and 5,6-dihydroxyindole-2-carboxylic acid (DHICA), have shown that DHI melanin are black in color on account of an intense absorption over the visible region, while DHICA melanins are lighter in color, exhibiting an intense absorption in the UVB/UVA range, the most dangerous portion of the solar spectrum, and a moderate absorption in the visible region, together with a marked antioxidant activity [12,13,14,15]. These features would point to the exploitation of DHICA pigments as active ingredients in dermo-cosmetic formulations with a combined sunscreen action and protection toward cellular damages induced by UV light and oxidative stress [16,17].

Based on these considerations, we recently explored the possibility to tune the properties of DHICA melanins for dermo-cosmetic purposes through modification at the carboxylic group. Melanins from DHICA methyl ester showed remarkable antioxidant properties in conventional chemical assays and in lipid peroxidation inhibition together with an improved solubility profile with respect to DHICA melanin [18,19] and good cytocompatibility toward HaCaT cells [20]. Yet, these melanins, which on mass spectrometric analysis appeared as a collection of low molecular weight oligomers, were very light in color (pale yellow), with null absorption in the visible region, and expectedly even less effective in photoprotection than DHICA melanin. As alternative functionalization, amidation appeared a viable option, as amides are generally stable under physiological conditions, and in addition, the electron-donor character of the carboxyamide group was expected to favorably affect the chromophore of the final melanin pigment with respect to carbomethoxy group. To this aim, we recently developed a straightforward and general procedure for the synthesis of DHICA carboxy amides that allowed to obtain the starting materials for pigment synthesis on the gram scale [21]. A preliminary analysis of the oxidation behavior of DHICA carboxybutanamide (ADHICA) in air at pH 9 showed a rapid melanization of the compound with the formation of a pigment whose spectral features over the UV-Vis spectrum were typical of a melanin [5,13]. On this basis, we report herein a complete structural characterization of the ADHICA melanin by UV-Vis, EPR, ATR-FTIR spectroscopy and MS analysis. Moreover, the solubility properties in selected solvents of dermo-cosmetic relevance and inhibition activity of photo- and radical-induced lipid peroxidation of this pigment were evaluated in comparison to DHICA melanin.

## 2. Results and Discussion

### 2.1. Oxidation of ADHICA

ADHICA was prepared by amidation of DHICA in the *O*-acetyl protected form in the presence of a coupling reagent, as recently reported [21].

In a first series of experiments, the oxidative chemistry of ADHICA was preliminarily explored to assess whether the insertion of a long aliphatic chain via an amide bond could affect the polymerization pattern observed with DHICA. To this aim, ADHICA was subjected to the copper-assisted aerobic oxidation under the conditions previously optimized for DHICA leading to the preferential formation of the 4,4′ dimer that is at a 3 mM concentration of the indole in the presence of equimolar copper acetate in HEPES buffer pH 7.5 for 10 min [5,22].

**Figure 1:**
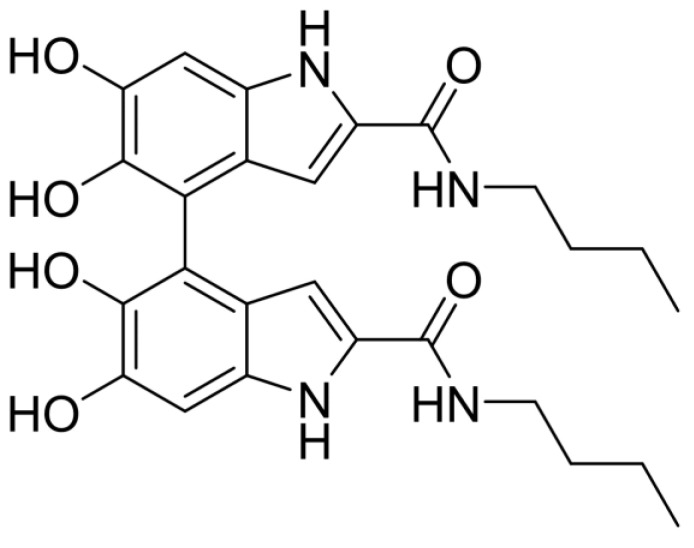
Structure of the 4,4′ dimer of ADHICA.

HPLC analysis of the reaction mixture after acetylation treatment (Figure S1) indicated formation of a main dimeric compound (LC-MS evidence) eluted at ca. 14 min, together with a minor product at R_t_ = 19 min. Subsequently, the main product was isolated in ca. 76% yield by preparative HPLC (Figure S2) and, further, to a complete mono- and bidimensional NMR analysis (Figures S3 and S4), it was formulated as the 4,4′-dimer (Figure 1) also by comparison with the data previously reported for DHICA dimers [22]. Of diagnostic value is the shielded proton resonance at 6.60 ppm, a feature characteristic of the 4,4′ arrangement. This result confirmed that the regioselectivity of the dimerization reaction observed for DHICA is not affected by amidation of the carboxyl function.

### 2.2. Preparation and Structural Characterization of ADHICA Melanin

Melanin from ADHICA was prepared by aerobic oxidation with the indole at 1 mM in 0.05 M sodium carbonate at a pH value of 9.0, close to the pKa of the catechol systems, to have the more oxidizable ionized form present in the mixture. For comparative purposes, also, a DHICA melanin was prepared under the same conditions. After 24 h, a significant lowering of the absorption maximum of the starting indole at λ = 320 nm was observed, while a broad featureless absorption in the whole UV-Vis range increased (Figure S5), typically associated with the melanization process. The reaction mixture was then acidified to pH 3, and the precipitated melanin was recovered by centrifugation and subsequent lyophilization. It should be noted that the conditions chosen for preparation of this melanin are quite different from those of our previous studies, in which a 10 mM concentration of the indole was used [18]. This is to allow for a more rapid consumption of the starting indole that decayed completely after 24 h oxidation in air. On the other hand, the recovery yields of the melanin proved satisfactory (>90% for ADHICA melanin).

EPR analysis of ADHICA melanin showed the broad singlet typical of eumelanins, see Figure S6, with a g-factor value indicating the prevalence of carbon centered radicals. Overall, the EPR features of ADHICA and DHICA melanins were very similar (see Table 1), even though some peculiarities of the ADHICA pigment could be highlighted. As an example, its spin density was lower than that measured for DHICA melanin but still in line with the values reported for natural and synthetic melanins [23]. The low gaussian fraction in ADHICA melanin pointed to the presence of resonant radicals all presenting a similar chemical nature. The DB value observed for ADHICA is slightly larger than that observed for DHICA melanin, which is in substantial agreement with the values previously reported for DHICA melanin prepared under different oxidation conditions [24], suggesting increased dipolar interaction of unpaired magnetic moments of free radicals located at short distances. The lower number of spins, located on the average at shorter distance, points to a slightly less homogeneous distribution of the radical centers within ADHICA with respect to DHICA melanin. This is confirmed by the power saturation profiles, which are very similar, but still allow to appreciate that ADHICA pigment shows a slightly less homogeneous relaxation behavior (see Figure S6).

ATR-FTIR spectra of both ADHICA and DHICA melanins were characterized by the presence of a broad band (Band 1) at 3500–3000 cm^−1^ (OH stretching). A Band 2 at 1685 cm^−1^ was observed for DHICA melanin, typically associated with the COOH carbonyl stretching, while ADHICA melanin showed the characteristic amide I and II bands at 1650 cm^−1^ (CONH carbonyl stretching) and 1520 cm^−1^ (NH bending), indicated as Band 3 and Band 4 in Figure S7. In addition, a Band 5 at 1260 cm^−1^ and 1445 cm^−1^, attributable to the bending of CH_3_ and CH_2_ of the aliphatic chain of ADHICA, were observed (Figure S8).

Finally, UV-Vis analysis of ADHICA melanin vs. DHICA melanin was performed at a concentration (0.01 mg/mL in methanol) that allowed full solubilization of either pigment (Figure S8). Notably, the absorbance of DHICA and ADHICA melanin are comparable in all visible ranges and even higher in the case of ADHICA melanin in the UVA 300–400 nm range, of particular interest for dermo-cosmetic application.

HPLC analysis of ADHICA melanin subjected to acetylation treatment and solubilized in methanol at 1 mg/mL provided evidence for the presence of a variety of species all eluting at higher retention times with respect to the 4,4′ dimer, likely oligomers up to tetramers (Figure S9), as indicated by LCMS analysis. The presence of oligomers up to tetramers was also confirmed by MALDI MS analysis, which, however, failed to provide evidence for higher oligomers due to difficulties in obtaining an efficient ionization of the samples in spite of different matrices and analytical conditions tested.

### 2.3. Solubility Properties of ADHICA Melanin in Solvents of Dermo-Cosmetic Interest

Based on the preliminary evaluation [21], the solubility properties of ADHICA melanin in different solvents of relevance for dermo-cosmetic applications were investigated.

**Figure 2:**
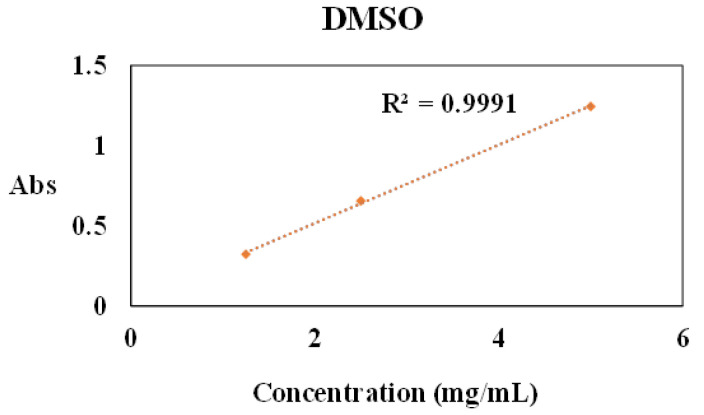
Absorbance versus concentration plots for ADHICA melanin in DMSO as the solvent.

To this aim, ADHICA melanins were added to methanol, DMSO, ethanol, propylene glycol and propylene glycol/glycerol 1:1 *v*/*v* at different doses (0.25–5 mg/mL), and the resulting mixtures were taken under stirring for 15 min at room temperature. After centrifugation, the UV–Vis spectra of the supernatants were recorded following appropriate dilution (1:100). The absorbance versus concentration curves were plotted to quantify the maximum solubility for each solvent that is reported in Table 2. For all plots, the maximum concentration in which the linearity of the absorbance is observed was determined, while, at higher doses, the linearity was lost (see Figure S10). Notably, the highest solubility, up to 5 mg/mL, was observed for DMSO (Figure 2).

### 2.4. Antioxidant Properties of ADHICA Melanin

Two widely used chemical assays were performed for the evaluation of the antioxidant properties of ADHICA melanin in comparison with DHICA melanin, namely the 2,2-diphenyl-1-picrylhydrazyl (DPPH) assay, measuring the hydrogen- and/or electron-donor ability of a given species, and the ferric-reducing antioxidant power (FRAP) test, measuring the iron (III)-reducing power. For either melanin, a stock solution in DMSO at 0.5 mg/mL was prepared to run the assays. As shown in Table 3, ADHICA melanin showed an EC_50_ value five times lower than that obtained for DHICA melanin, indicating a noticeable higher antioxidant efficacy as the H donor of this pigment. On the other hand, in the FRAP assay, the two melanins exhibited comparable activities.

These results should be interpreted primarily in the light of the different solubility properties of ADHICA and DHICA melanins in the assay media. In the case of the DPPH assay, the use of ethanol as the solvent is expected to favor ADHICA melanin. Yet, the noticeable increase of the antioxidant power observed would indicate that, indeed, this melanin is endowed with a higher activity with respect to DHICA melanin, which is not merely due to the solubilization in the assay medium. Note that, in the concentration range explored to determine the EC_50_ value (that is, 0.005–0.286 mg/mL), DHICA melanin also previously dissolved in DMSO shows an acceptable solubility in ethanol. This would therefore suggest that the presence of the amide group significantly improves the antioxidant properties of the pigment. On the other hand, both melanin performed comparably in the FRAP assay, indicating that ADHICA exhibited a comparable Fe (+3) reduction ability with respect to DHICA melanin.

To evaluate the ability of ADHICA melanin to prevent the oxidation of critical biological targets, the effect on the peroxidation of linoleic acid as a model system of lipid components of cellular membranes was tested in comparison with DHICA melanin.

The reaction was carried out using a radical initiator that is 2,2′-azobis(amidinopropane) dihydrochloride (AAPH) in aqueous solution at pH 7.4 in the presence or absence of melanin (0.02–0.04 mg/mL). The peroxidation process was followed by monitoring conjugated diene hydroperoxide formation at 234 nm. In this case, the EC_50_ value obtained for ADHICA melanin was half that determined for DHICA melanin (Table 3).

In other experiments, linoleic acid oxidation was induced by exposure to sunlight in the presence of riboflavin as a photosensitizer [25]. Additionally, in this case, ADHICA melanin showed a relatively low EC_50_ value, whereas, in the same range of concentrations explored, DHICA melanin did not show appreciable activity (Table 4). The effects of ADHICA are likely due to synergistic effect of UV-Vis screening and ability to inhibit the propagation chain in the peroxidation process.

## 3. Materials and Methods

3,4-dihydroxy-L-phenylalanine (L-DOPA), potassium ferricyanide, sodium bicarbonate, sodium dithionite, N,N-diisopropylethylamine (DIPEA), anhydrous dimethylformamide (DMF), 1-[Bis(dimethylamino) methylene]-1*H*-1,2,3-triazolo [4,5-b]pyridinium-3-oxide hexafluorophosphate (HATU), 1-butanamine, pyridine, acetic anhydride, copper acetate, 4-(2-hydroxyethyl)-1-piperazineethanesulfonic acid (HEPES), linoleic acid, 2,2-diphenyl-1-picrylhydrazyl (DPPH), 2,2′-azobis(-amidinopropane)dihydrochloride (AAPH), sodium dodecyl sulfate (SDS), 2,4,6-tris(2 -pyridyl)-s-triazine and 6-hydroxy-2,5,7,8-tetramethylchroman-2-carboxylic acid (Trolox) were purchased by Sigma-Aldrich (Milan, Italy). All solvents were HPLC grade. Bi-distilled deionized water was used throughout the study. DHICA and ADHICA were prepared as described [5,21].

UV-Vis spectra were recorded on a Jasco V-730 Spectrophotometer (Cremella, Lecco, Italy).

NMR spectra were recorded at 400 MHz on a Bruker instrument (Milan, Italy).

Electron paramagnetic resonance (EPR) measurements were performed using a Bruker Elexys E-500 spectrometer (Milan, Italy) equipped with a superhigh sensitivity probe head. The analyses were carried out under conditions previously described [26]. Since sample hydration was not controlled during the measurements, spin density values have to be considered as order of magnitude estimates [27].

MALDI mass spectrometric analyses were run on an AB Sciex TOF/TOF 4800 instrument using 2,5-dihydroxybenzoic acid as the matrix, and the melanin were applied to the plate from a fine suspension in various solvents (DMSO and MeOH) at different concentrations (1, 2 and 5 mg/mL) with or without acetylation treatment. Different spectrums represent the sum of 4000 laser pulses from randomly chosen spots per sample position. Raw data were analyzed using the computer software provided by the manufacturers and are reported as monoisotopic masses.

ATR-FTIR spectra were recorded on a Nicolet 5700 Thermo Fisher Scientific instrument (Milan, Italy). Spectra were recorded as an average of 128 scans in the range 4000−450 cm^−1^ (resolution of 4 cm^−1^).

HPLC analysis was performed with an instrument equipped with a UV-Vis detector (Agilent, G1314A, Cernusco sul Naviglio, Milan, Italy); a Phenomenex (Castel Maggiore, Bologna, Italy) Sphereclone ODS column (250 × 4.60 mm, 5 µm) was used at a flow rate of 0.7 mL/min; a 0.1% formic acid (solvent A)/methanol (solvent B) gradient elution was performed as follows: 50% B, 0–5 min; from 50 to 70% B, 5–45 min; the detection wavelength was 300 nm. Preparative HPLC was performed coupled with a UV spectrophotometer (Cernusco sul Naviglio, Milan, Italy) set at 300 nm; an Econosil (Castel Maggiore, Bologna, Italy) C18 column (10 μm, 22 × 250 mm) was used at a flow rate 25 mL/min; Eluent system: 0.1% formic acid (solvent A)/methanol (solvent B) in a 40:60 ratio, respectively.

LC-MS analysis was performed on an Agilent ESI-TOF 1260/6230DA instrument (Cernusco sul Naviglio, Milan, Italy) in positive ion mode in the following conditions: nebulizer pressure 35 psig; drying gas (nitrogen) 5 L/min, 325 °C; capillary voltage 3500 V; fragmentor voltage 175 V. An Eclipse Plus (Agilent, Cernusco sul Naviglio, Milan, Italy) C18 column, 150 × 4.6 mm, 5 μm, at a flow rate of 0.4 mL/min was used, with 0.1% formic acid:methanol 40: 60 *v*/*v* as the eluant.

### 3.1. Oxidation of ADHICA and Isolation of Its 4,4′-Dimer

Oxidation of ADHICA was run following a procedure previously reported with slight modifications [22]. ADHICA (245 mg, 3 mM) and 1 molar eq of copper acetate (197 mg) were dissolved in 0.1 M HEPES buffer (329 mL, pH 7.5), and the mixture was stirred for 10 min. The reaction was stopped by addition of 40 mg of sodium dithionite and acidification to pH 3 with 6 M HCl. The mixture was extracted with ethyl acetate (4 × 200 mL), and the combined organic phases were filtered on anhydrous sodium sulphate and taken to dryness at a rotary evaporator. The residue (169 mg) was treated with 2 mL of acetic anhydride and 100 μL of pyridine overnight. After removal of the solvents, the acetylated mixture was dissolved in DMSO and purified by preparative HPLC to give the 4,4′-dimer of ADHICA in its acetylated form (150 mg, 75% yield).

ES-MS: *m*/*z:* 663 (M+H)^+^, 685 (M+Na)^+^, 701 (M+K)^+^.

^1^H-NMR (DMSO-d_6_): δ (ppm): 0.85 (3H × 2, t, J = 6.0 Hz), 1.25 (2H × 2, m), 1.43 (m, 2H × 2, quint., J = 6.0 Hz), 1.95 (3H × 2, s), 2.28 (3H × 2, s), 3.18 (2H × 2, m), 6.60 (1H × 2, bs), 7.35 (1H × 2, bs), 8.39 (1H × 2, t, J = 4.8 Hz), 11.83 (1H × 2, bs).

^13^C-NMR (DMSO-d_6_): δ (ppm): 14.08 (CH_3_), 20.01 (CH_2_), 20.32 (CH_3_), 20.97 (CH_3_), 31.56 (CH_2_), 38.81 (CH_2_), 102.80 (CH), 106.83 (CH), 125. 04 (C), 133.68 (C), 134.76 (C), 139.87 (C), 160.77 (C), 168.53 (C), 169.14 (C).

### 3.2. Preparation of Melanins from DHICA and ADHICA

Melanins were prepared by aerial oxidation of DHICA or ADHICA (1 mM) in 0.05 M carbonate buffer at pH 9.0 under stirring. After 24 h, a precipitate was collected by acidification of the reaction mixture to pH 3 and centrifugation (7000 rpm, 10 min, 4 °C). The sample was washed three times with 0.01 M HCl (15 mL) and then lyophilized. DHICA and ADHICA melanin were obtained in 85 and 95 *w*/*w* yield, respectively.

### 3.3. Evaluation of the Solubility Properties of DHICA and ADHICA Melanins

DHICA or ADHICA melanin were added to methanol, DMSO, ethanol, glycerol and propylene glycol/glycerol 1:1 *v*/*v* in different amounts (0.25–5 mg/mL), and the resulting mixtures were taken under stirring for 15 min at room temperature. After centrifugation (5000× *g*, 10 min, 4 °C), the UV-Vis spectra of the supernatants were recorded following appropriate dilution (1:100).

### 3.4. DPPH Assay

To a 0.2 mM ethanolic solution of DPPH, DHICA or ADHICA melanin was added (concentration range 0.005–0.286 mg/mL), and after 10 min under stirring at room temperature, the absorbance of the solution at 515 nm was measured. Experiments were run in triplicate [28].

### 3.5. FRAP Assay

To 0.3 M acetate buffer (pH 3.6) containing 1.7 mM FeCl_3_ and 0.83 mM TPTZ, DHICA or ADHICA melanins were added (final dose 0.005–0.03 mg/mL), and after 10 min under stirring at room temperature, the absorbance of the solution at 593 nm was measured. Results were expressed as Trolox equivalents (eqs). Experiments were run in triplicate [29].

### 3.6. Inhibition of Lipid Peroxidation

A procedure previously reported was followed with minor modifications [30]. In brief, 0.25 mL of linoleic acid solution, prepared dissolving 22 mg in 5 mL of acetonitrile, was added dropwise to 5 mL of 0.05 M borate buffer (pH 9.0), containing 0.25 mL of Triton X-100. The resulting dispersion was clarified by adding 1 mL of 1 M sodium hydroxide. The volume was adjusted to 50 mL with an additional borate buffer. The resulting linoleic acid solution (16 mM) was stored at 4 °C in the dark until needed. A solution of AAPH at 40 mM was freshly prepared in 0.05 M phosphate buffer (pH 7.4).

Additionally, 30 μL of the 16 mM linoleic acid dispersion was added to the UV cuvette containing 2.80 mL of 0.05 M phosphate buffer (pH 7.4), thermostated prior at 37 °C. The oxidation reaction was initiated at 37 °C under air by the addition of 150 μL of 40 mM AAPH solution. Lipid peroxidation was carried out in the presence of a proper amount of a solution of DHICA melanin or ADHICA melanin in DMSO. The rate of oxidation at 37 °C was monitored by recording the increase in absorption at 234 nm due to the formation of conjugated diene hydroperoxides. AAPH has a relatively high absorbance below 260 nm, which decreases as the compound decomposes. Therefore, its absorbance measured separately in the absence of linoleic acid but, in the presence of melanin, was subtracted from each experimental point.

Photoinduced lipid peroxidation was carried out on a solution of linoleic acid at 2.5 mM concentration in 0.1 M phosphate buffer at pH 7.0 containing sodium dodecyl sulfate (SDS) at a final concentration of 0.05 M in the presence of riboflavin as photosensitizer at 20 µM concentration and melanin solution at a concentration in the range of 0.02–0.05 mg/mL.

The solutions were exposed to sunlight in July from 12:00 to 12.30 in the southwest of Italy (40°50′19.81″ N 14°11′06.04″ E). The absorbance at 234 nm of solutions containing riboflavin and melanins, in the absence of linoleic acid, was subtracted from each experimental point.

No significant formation of conjugated diene hydroperoxides was observed in the control, non-irradiated linoleic acid solutions with and without riboflavin, ruling out the occurrence of autooxidation processes.

## 4. Conclusions

The search for natural or naturally derived products as functional ingredients of dermo-cosmetic formulations is nowadays very active to respond to consumers’ increasing demand for products that might ensure high activity even at low doses while not raising health hazard issues. Of particular interest in this connection are eumelanins that are renowned for their antioxidant potential and photoprotective role [9]. Yet, the high structural variability and low availability from natural sources have directed the research activity toward melanin-related pigments prepared from the indole precursors by biomimetic low-cost procedures. In this frame, the present work has explored the potential of a melanin obtained from an amide derivative of the major melanin precursor DHICA showing how the introduced functionalization allows to improve the solubility of the final pigment in polar organic solvent of dermo-cosmetic interest while maintaining or even ameliorating the favorable properties of the pigments prepared from DHICA, i.e., the intense absorption in the UVA and all the visible spectrum, as well as the antioxidant activity. Indeed, structural investigation and analysis of the early oligomers indicated that this pigment is substantially similar to DHICA melanin with respect to the regiochemistry of the oxidative coupling. This finding, along with the similar paramagnetic properties and spin density of the pigments, hints to a comparable distribution of oxidized/reduced dihydroxyindole units. Differently from the melanin from the methyl ester of DHICA that showed very good antioxidant properties, but poor covering of the visible spectrum [10], ADHICA melanins, thanks to the electrodonating features of the amide functionality, showed an absorption in the UV-Vis region even higher than DHICA melanin and proved also more active against radical and light induced lipid peroxidation, suggesting the potential of this pigment in counteracting the degradation of unsaturated fatty acid components of triglycerides and membrane phospholipids associated with inflammatory conditions. When considered also in the light of the streamlined procedure for getting the amide monomer on gram scale recently developed [13], the ADHICA melanin may be regarded as a promising dermo-cosmetic ingredient provided that the toxicity profile will encourage further studies to assess its actual effectiveness, e.g., in antiaging, anti-inflammatory, sunlight photoprotective formulations.

## Figures and Tables

**Table 1:** EPR data for DHICA and ADHICA melanin.

Samples	Spin/g Concentration	ΔB (±0.2)	Gauss Fraction	g-Factor (±0.0003)
DHICA melanin	1.9 × 10^18^	3.9 ± 0.2	0.47	2.0029 ± 0.0003
ADHICA melanin	4.8 × 10^17^	4.7 ± 0.2	0.28	2.0030 ± 0.0003

**Table 2:** Solubility properties of ADHICA melanin.

Solvent	Maximum Solubility (mg/mL)
DMSO	5
Ethanol	1.5
Methanol	1
Propylene glycol	0.33
Propylene glycol/glycerol	0.25

**Table 3:** EC_50_ values in DPPH and FRAP assays for DHICA and ADHICA melanins. Reported are the mean ± SD values of at least three experiments.

Samples	EC_50_ (mg/mL)	Trolox eqs. (mg/mL)
DHICA melanin	0.242 ± 0.004	0.33 ± 0.01
ADHICA melanin	0.0374 ± 0.0004	0.246 ± 0.004

**Table 4:** Chemical and photo-induced lipid peroxidation assay in the presence of DHICA and ADHICA melanin.

	DHICA Melanin mg/mL	ADHICA Melanin mg/mL
EC_50_ (AAPH)	0.0598 ± 0.0008	0.037 ± 0.004
EC_50_ (solar light)	Not detectable	0.062 ± 0.001

## Data Availability

Not applicable.

## Supplementary Materials

The following supporting information can be downloaded at: https://www.mdpi.com/article/10.3390/ijms24044241/s1.

## Abbreviations

AAPH	2,2′-azobis(amidinopropane) dihydrochloride
ADHICA	5,6-dihydroxyindole-2-carboxybutanamide
DHI	5,6-dihydroxyindole
DHICA	5,6-dihydroxyindole-2-carboxylic acid
DIPEA	N,N-diisopropylethylamine
DMF	dimethylformamide
DMSO	dimethyl sulfoxide
DOPA	3,4-dihydroxy-L-phenylalanine
DPPH	2,2-diphenyl-1-picrylhydrazyl
FRAP	Ferric Reducing Antioxidant Power
HATU	1-[Bis(dimethylamino) methylene]-1H-1,2,3-triazolo [4,5-b]pyridinium-3-oxide hexafluorophosphate
HEPES	4-(2-hydroxyethyl)-1-piperazineethanesulfonic acid
SDS	sodium dodecyl sulfate
TPTZ	2,4,6-Tris(2-pyridyl)-s-triazine
Trolox	6 -hydroxy-2,5,7,8 -tetramethylchroman-2-carboxylic acid
