# Supplementary material for: A Model Eumelanin from 5,6-Dihydroxyindole-2-Carboxybutanamide Combining Remarkable Antioxidant and Photoprotective Properties with a Favourable Solubility Profile for Dermo-Cosmetic Applications
Source: Int J Mol Sci. 2023 Feb 20;24(4):4241. doi: 10.3390/ijms24044241 (PMC9961549; doi:10.3390/ijms24044241)
Supplement: Supplementary file 1 [file ijms-24-04241-s001.zip › ijms-2206723-supplementary.pdf]

Supporting Information

A Model Eumelanin from 5,6-Dihydroxyindole-2-Carboxybutanamide Combining Remarkable Antioxidant and Photoprotective Properties with a Favourable Solubility Profile for Dermo-Cosmetic Applications

Table of contents	
**Figure S1.** HPLC profile of acetylated ADHICA oxidation mixture in the presence of Cu^{2+}.	p. 1
**Figure S2.** HPLC profile of the main product of copper assisted ADHICA oxidation after purification by preparative HPLC.	p. 2
**Figure S3.** ^{1}H-NMR spectrum of acetylated ADHICA 4,4'-dimer (DMSO-d_6).	p. 3
**Figure S4.** ^{13}C-NMR spectrum of acetylated ADHICA 4,4'-dimer (DMSO-d_6).	p. 4
**Figure S5.** UV-Vis spectra of the aerobic oxidation mixture of 1 mM ADHICA in carbonate buffer at pH 9.0 at different reaction times	p. 5
**Figure S6.** a) Solid state EPR spectra and b) power saturation profiles of DHICA and ADHICA melanin.	p. 6
**Figure S7.** FTIR-ATR spectra of **ADHICA** (red line) and **DHICA** (black line) melanin.	p. 7
**Figure S8**. UV-Vis spectrum of ADHICA and DHICA melanins at 0.01 mg/mL in methanol.	p. 8
**Figure S9.** HPLC profile of the ADHICA melanin at 1mg/mL in DMSO. Detection wavelength at 300 nm.	p. 9
**Figure S10.** Absorbance versus concentration plots for ADHICA melanin in various solvents. Correlation coefficients for linearity fitting among different data points **Orange line** ($R^2 > 0.99$), **Blu line** ($R^2 < 0.99$).	p. 10

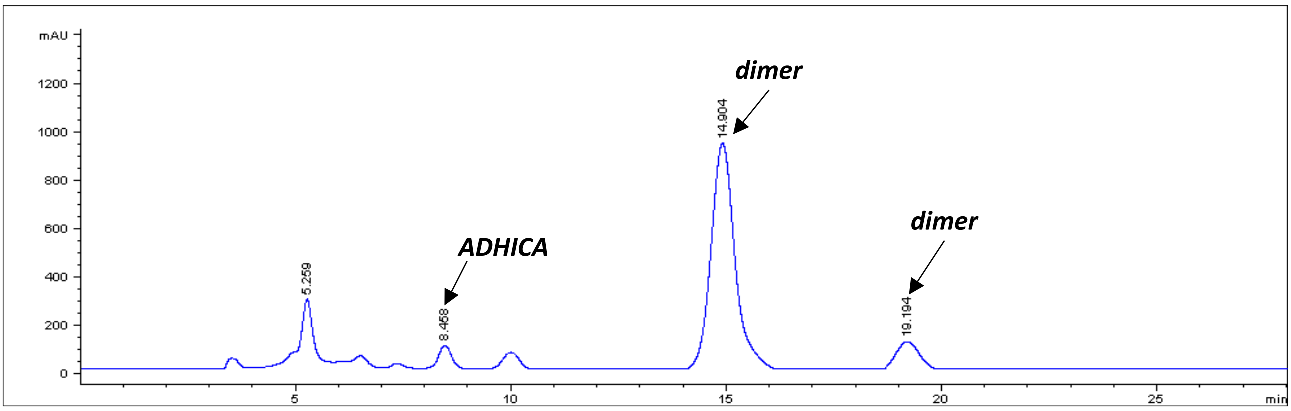

Figure S1. HPLC profile of acetylated ADHICA oxidation mixture in the presence of Cu^{2+}.

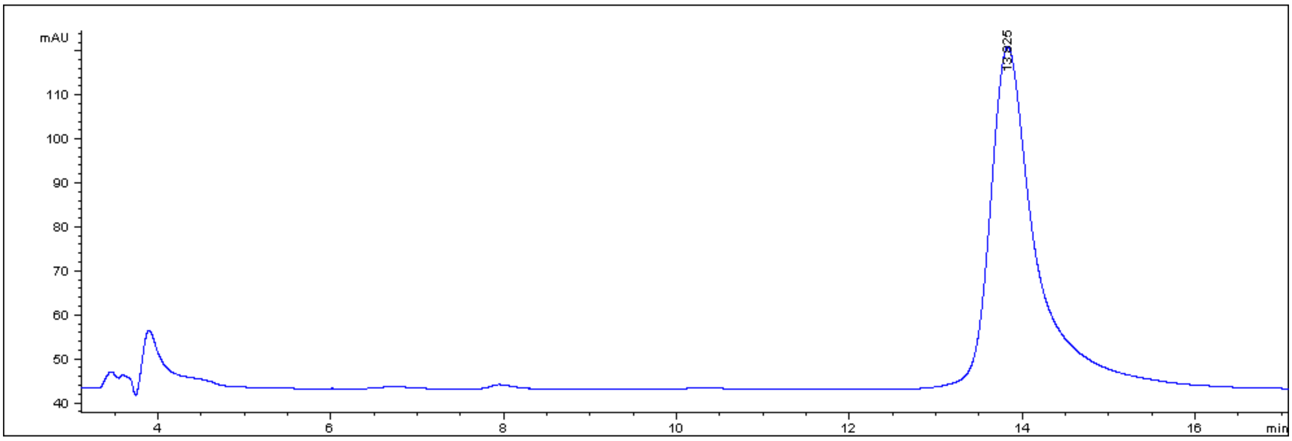

Figure S2. HPLC profile of the main product of ADHICA oxidation after purification by preparative HPLC.

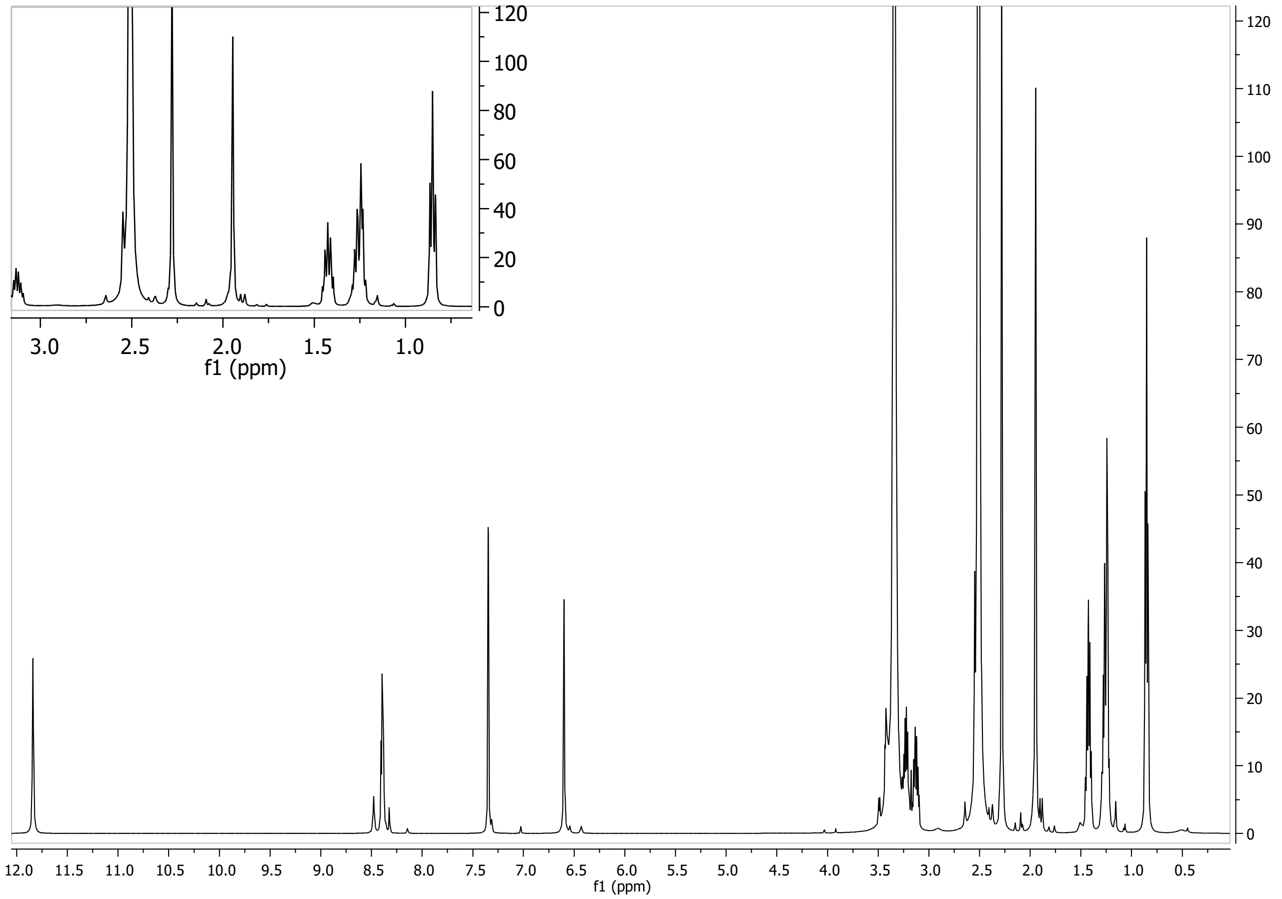

Figure S3. ^{1}H-NMR spectrum of acetylated ADHICA 4,4-dimer (DMSO-d_6).

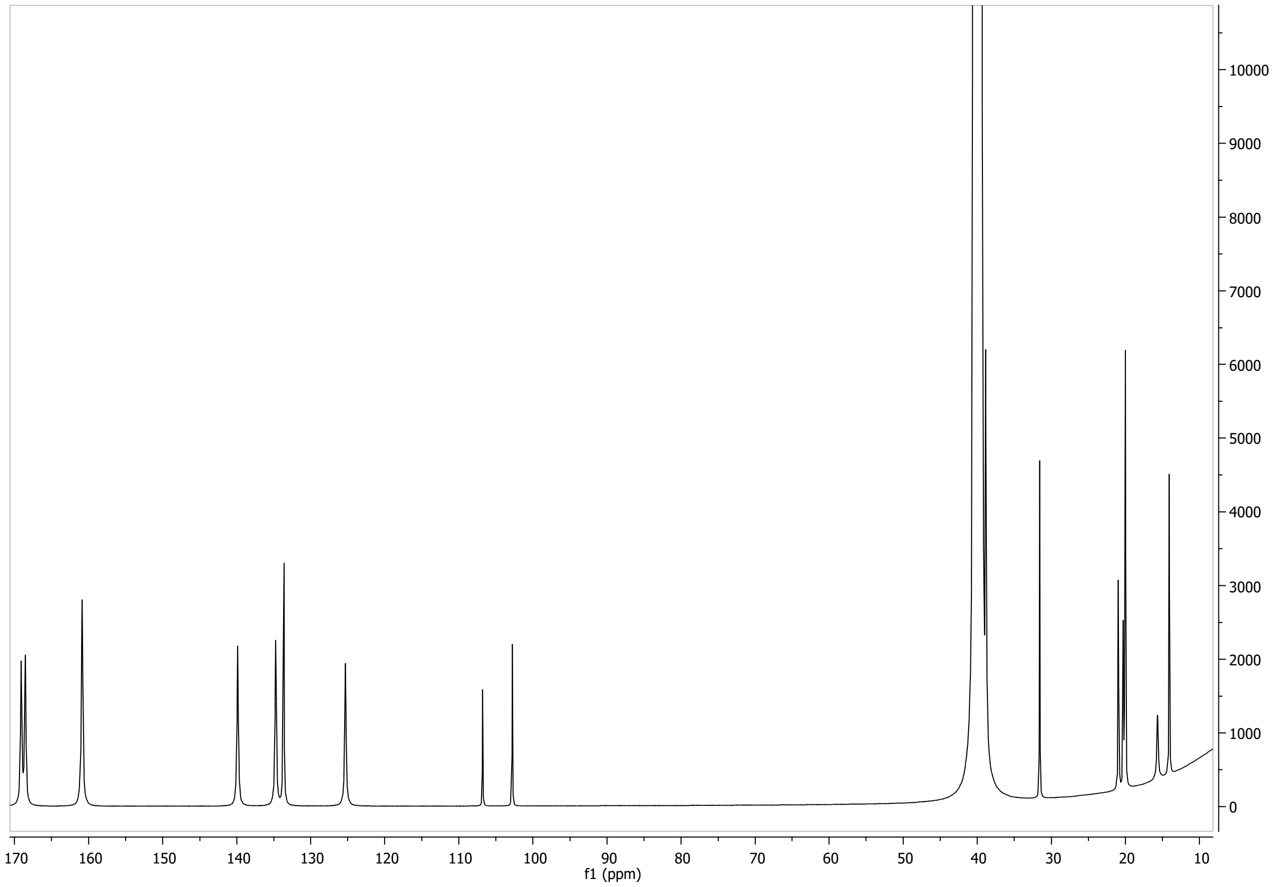

Figure S4. ^{13}C-NMR spectrum of acetylated ADHICA 4,4'-dimer (DMSO-d_6).

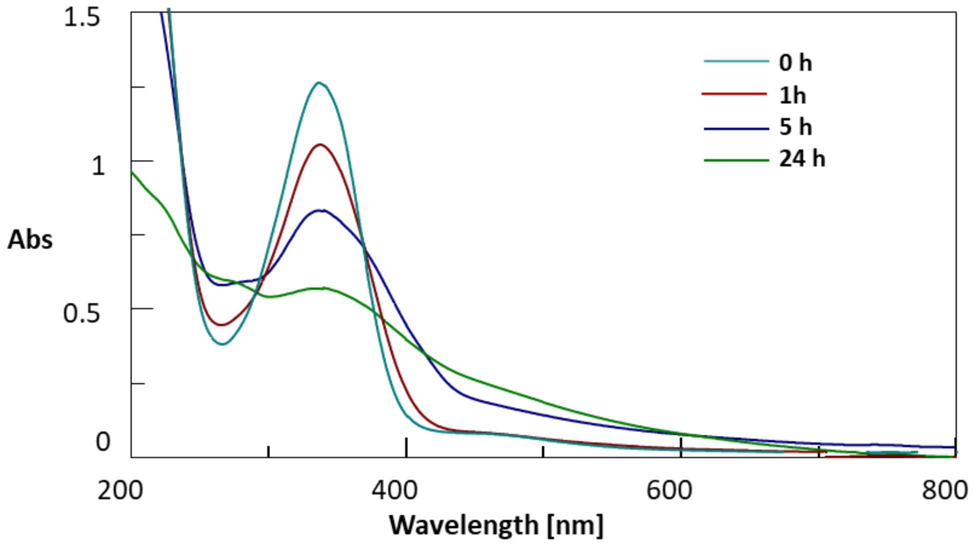

Figure S5. UV-Vis spectra of the aerobic oxidation mixture of 1 mM ADHICA in carbonate buffer at pH 9.0 at different reaction times.

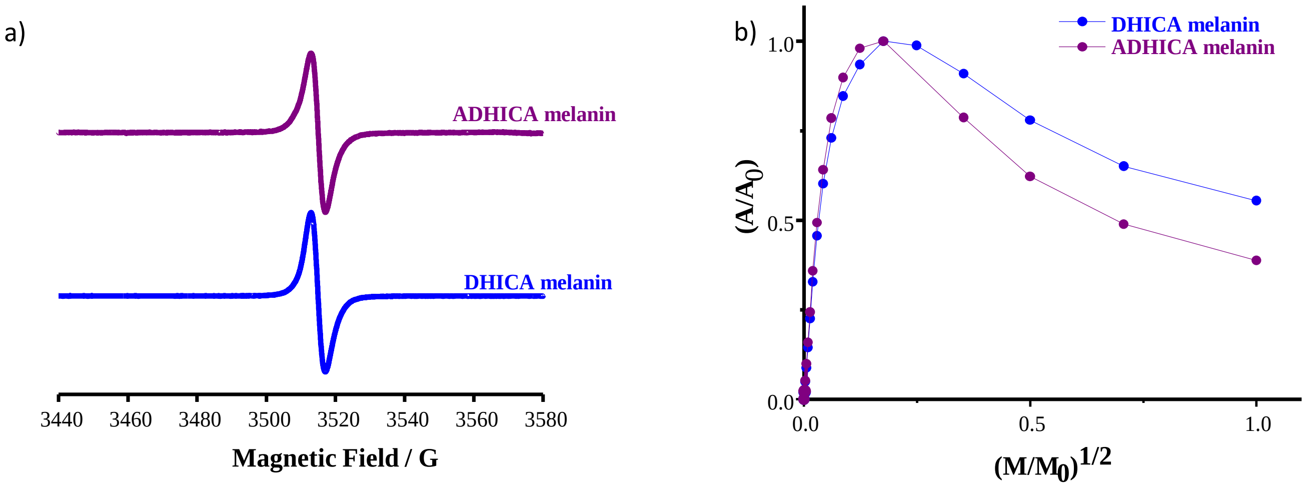

Figure S6. a) Solid state EPR spectra and **b**) power saturation profiles of DHICA and ADHICA melanin.

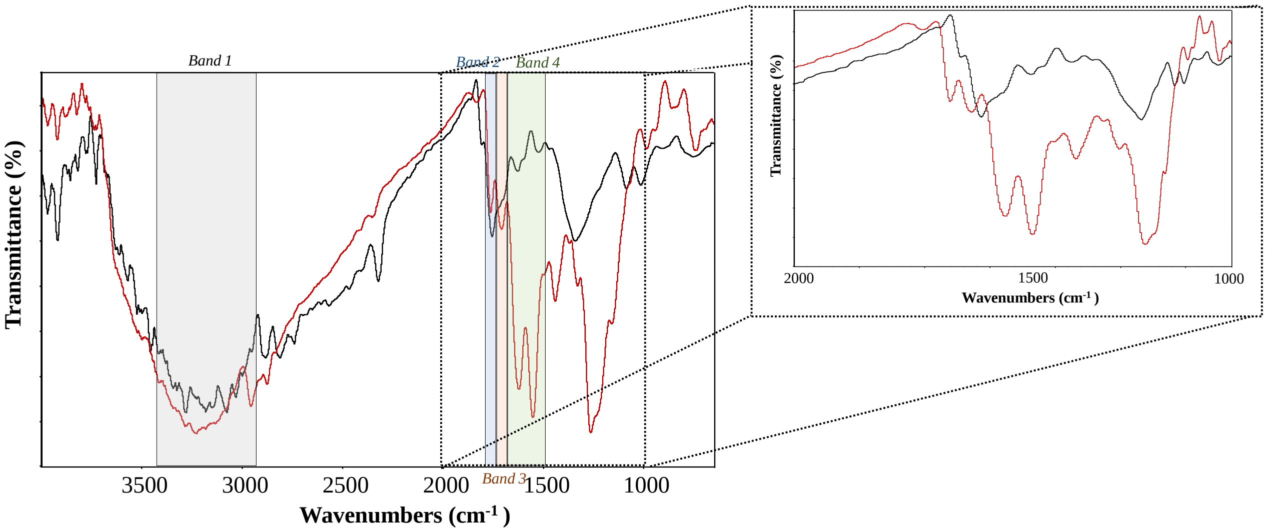

Figure S7. FTIR-ATR spectra of **ADHICA** (red line) and **DHICA** (black line) melanin.

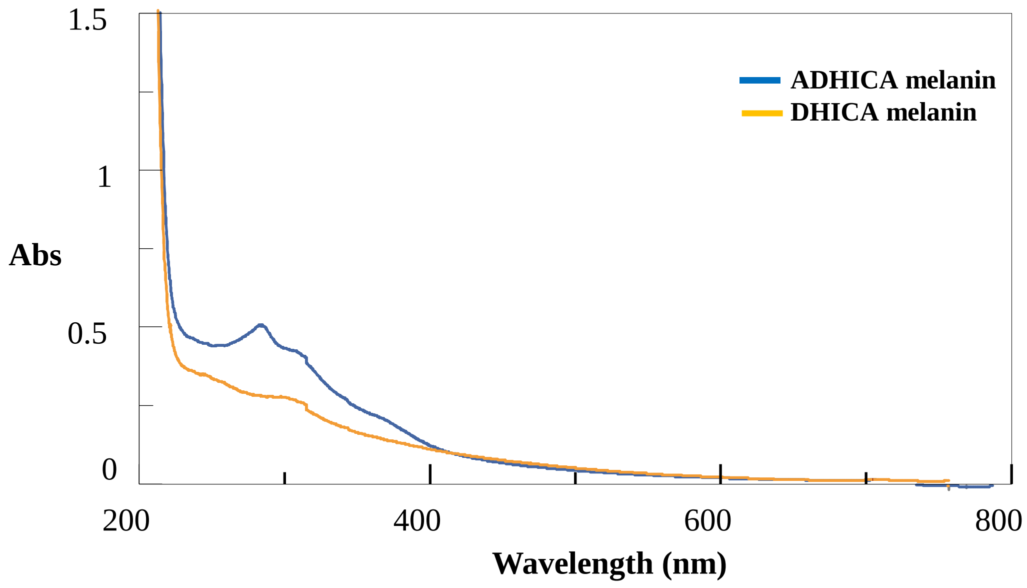

Figure S8. UV-Vis spectrum of ADHICA and DHICA melanin at 0.01 mg/mL in methanol.

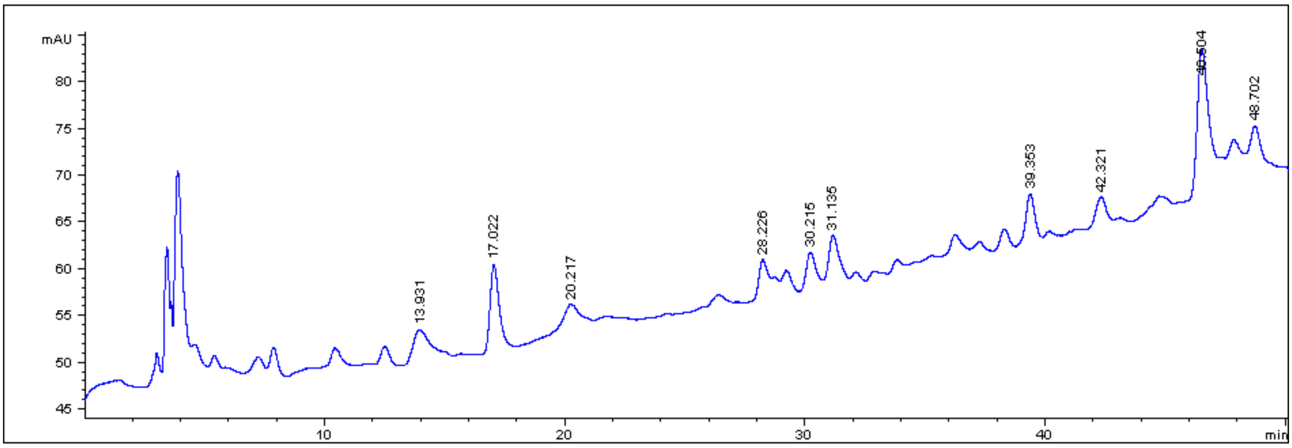

Figure S9. HPLC profile of the ADHICA melanin at 1mg/mL in DMSO. Detection wavelength at 300 nm.

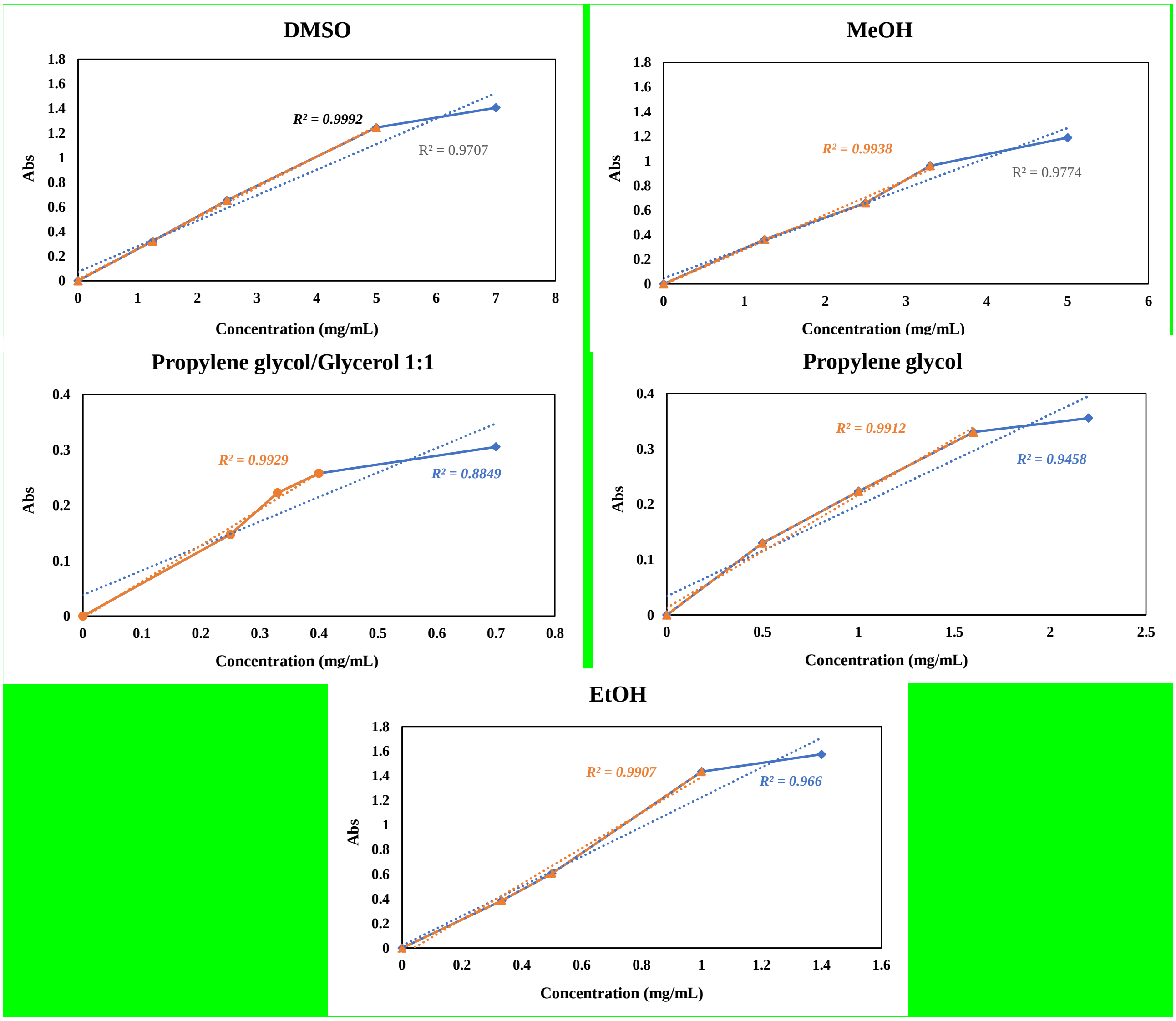

Figure S10. Absorbance versus concentration plots for ADHICA melanin in various solvents. Correlation coefficients for linearity fitting among different data points **Orange line** ($R^2 > 0.99$), **Blu line** ($R^2 < 0.99$).
